# Improving Mechanical and Corrosion Properties of 6061 Al Alloys via Differential Speed Rolling and Plasma Electrolytic Oxidation

**DOI:** 10.3390/ma17061252

**Published:** 2024-03-08

**Authors:** Siti Fatimah, Warda Bahanan, I Putu Widiantara, Jae Hoon Go, Jee-Hyun Kang, Young Gun Ko

**Affiliations:** Materials Electrochemistry Group, School of Materials Science and Engineering, Yeungnam University, Gyeongsan 38541, Republic of Korea

**Keywords:** Al alloys, differential speed rolling, plasma electrolytic oxidation, anti-corrosive coating, corrosion, hardness

## Abstract

This study explores the combined potential of severe plastic deformation (SPD) via differential speed rolling (DSR) and plasma electrolytic oxidation (PEO) to enhance the material performance of 6061 Al alloys. To this end, DSR was carried out at a roll-speed-ratio of 1:4 to obtain ~75% total thickness reduction and a final microstructure of <1 µm. The rest of the samples were annealed to obtain various grain sizes of ~1, ~25, and ~55 μm. Through DSR, the hardness of the material increased from ~64 to ~102 HV. Different grain sizes altered the plasma behavior which further influence the growth of the coating layer, where the fine grain size produced a compact structure beneficial for corrosion protection. This synergy offers tailored materials ideal for high-performance applications across diverse industries, combining enhanced bulk properties from DSR with optimized surface attributes from PEO.

## 1. Introduction

Aluminium (Al) and its alloys have been extensively utilized in a wide range of engineering sectors, including the aerospace, electronics, automotive, and architectural realms, primarily due to their advantageous properties of being lightweight yet possessing high specific strength. However, their extensive use has been limited by inherent drawbacks, notably their relatively low surface hardness and susceptibility to corrosion [1,2,3].

On the other hand, enhancing the grain structure’s refinement has emerged as a pivotal factor in reinforcing the mechanical properties of bulk Al alloys while preserving their inherent chemical composition [4,5,6]. Severe plastic deformation (SPD) imposes high amounts of strains involving complex stress or high shear, resulting in fine grain size without significant change in the dimensions of metal [4,6]. There are several types of SPD, including equal channel angular pressing (ECAP) and differential speed rolling (DSR) as the most known types. Among the methodologies employed for achieving grain refinement, severe plastic deformation approaches such as ECAP and DSR stand prominent. These techniques exert substantial shear and strain forces on sheet materials, facilitating a more effective attainment of finely grained structures [4]. However, fine-grained metals are more prone to corrosion due to their high-density grain boundaries. Therefore, surface treatment needs to be carried out to enhance their surface protective properties.

In recent years, significant attention has been directed towards plasma electrolytic oxidation (PEO) as an innovative surface treatment technique [1,2]. This technique has attracted significant attention due to its capacity to create protective coatings on valve metals such as Mg, Ti, and Al via an electrochemical process assisted by plasma utilizing the high potential of electric current [2]. These coatings exhibit superior mechanical properties and heightened resistance to corrosion when compared to the traditional anodization process [1,2].

There is a noticeable lack of comprehensive studies focusing on enhancing both the bulk and surface characteristics of lightweight alloys such as Al, Ti, and Mg, among others. This includes altering plasma behavior by considering the initial grain size. The inherent connection between grain size and the overall properties essential for structural applications remains inseparable. Therefore, a thorough investigation into plasma behavior during PEO, integrating an examination of grain size, holds significant potential as a valuable contributor to technology advancement across multiple engineering disciplines. Such research has the capacity to drive substantial progress applicable to diverse engineering applications.

The properties of metals and ceramics are correlated with their microstructures. Due to the demand for high-performance material, such metals should undergo various treatments. Among these techniques, grain refinement stands out as a pivotal approach for attaining heightened mechanical properties in sheet metal. According to Hall Petch eq., fine grain exhibits better mechanical properties compared to coarse grain which leads to an increase in the hardness value [4]. Grain refinement can be achieved through various techniques, with severe plastic deformation being one of the approaches commonly employed.

The role of grain size in the chemical response, especially corrosion properties, needs to be elaborated on due to intriguing and scarce results thus far. For instance, fine grain obtained from ECAP decreases the corrosion resistance of anodized Al-Mg alloy [7,8]. Moreover, fine-grained steel processed by advanced thermomechanical properties induces pitting corrosion [9]. On the other hand, fine grain samples processed by ECAP increases the corrosion resistance of PEO-coated Ti [10,11]. The coating growth mechanism during plasma electrolytic oxidation (PEO), considering the grain size, has never been the primary focus. Grain refinement in structural metals is very important for enhancing mechanical properties; hence, deep study into the role of grain size in the coating formation has also become important as it might facilitate the formation of anti-corrosion coatings. Therefore, the present study aims to scrutinize how initial grain size impacts the formation of the PEO coating and alters its properties.

## 2. Experimental Procedure

### 2.1. Differential Speed Rolling

**Figure 1:**
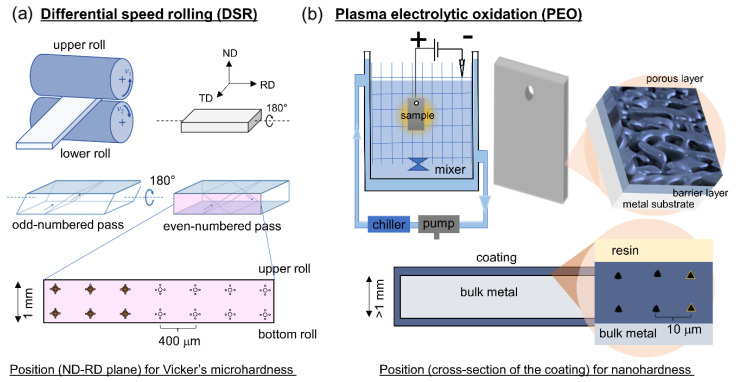
Schematic figures to illustrate (**a**) the differential speed rolling (DSR) process utilizing two identical rolls and (**b**) plasma electrolytic oxidation (PEO) in wet-based alkaline solution for the generation of a thick adherent coating. The bottom figures show how hardness measurements were conducted.

The initial substrate used in the present study was 6061 Al alloys with a chemical composition of 1.0 Mg, 0.6 Si, 0.28 Cu, 0.2 Cr, and balance Al (in wt.%). Initially, the samples were subjected to homogenization at a temperature of 550 °C for a period of 3 h. Following this, a gradual cooling process in ambient air facilitated the attainment of a fully annealed microstructure, characterized by an equiaxed grain size of approximately 45 μm. The sample was then cut into a plate with the size of 100 mm × 50 mm × 4 mm. A series of DSRs were performed using two rolls of the same size, each with a diameter of 210 mm, rotating at a speed ratio of 1:4 for the lower and upper rolls, respectively. This was conducted with the lower roll maintaining a constant velocity of approximately 3.4 m min^−1^. The samples experienced a 30% decrease in thickness during each pass, leading to an accumulative reduction of 75% following four consecutive DSR passes. The samples were subjected to a 180 rotation around their longitudinal axis between adjacent passes to maximize the shear bands for obtaining a fine grain structure. The grain size of the deformed sample was documented as about ~1 μm with a thickness of 1 mm, which was then labeled as SS. Further, the deformed samples underwent annealing treatment at temperatures of 550 °C and 560 °C for 10 h and 3 h, respectively, to achieve grain sizes of ~25 μm and ~55 μm which were then labeled as MS and LS, respectively. Figure 1a shows a schematic illustration of the DSR process and sample rotation utilized in the current work. Figure 1b illustrates the overall PEO process for obtaining a thick and adherent coating.

The mechanical properties of the Al alloy, deformed by DSR, underwent examination through hardness tests. This study utilized Vickers microhardness (Wilson Instrument, Norwood, MA, USA) testing with a 200 g load applied for 10 s to analyze the distribution of microhardness. Measurements were taken along a plane perpendicular to the rolling direction, following a grid pattern for indentation positioning. Microhardness values were determined by averaging measurements from four points surrounding the selected position, each spaced approximately 400 μm apart. On the other hand, the hardness of the coating was investigated through nanoindentation tests due to the limited coating thickness. The tests were conducted on a cross-section of the coating region as illustrated in Figure 1. The measurement was performed using a meticulously calibrated Nanovea Nanoindenter (Nanovea, Irvine, CA, USA), equipped with a three-sided diamond Berkovich indenter tip. In all nanoindentation measurements, a consistent loading rate of 0.2 N s^−1^ was employed while applying a maximum load of 2 N.

### 2.2. Plasma Electrolytic Oxidation

Prior to plasma electrolysis, the sample sheet, sized at 20 mm × 20 mm × 2 mm, underwent polishing by using #1200 grit SiC paper (ACP 1010, Gunpo, Republic of Korea). The base electrolyte selected for the experiment was a commonly employed alkaline phosphate solution, consisting of 5.6 g/L KOH and 2 g/L K_3_PO_4_. Plasma electrolysis was conducted by using a 20 kW AC power supply from Power Korea, outfitted with stirring and cooling systems, current density of 100 mA cm^−2^, and wave frequency of 60 Hz. The electrolyte featured a stainless-steel wire as the cathode and employed an Al alloy sample as its anode. The electrolyte temperature was modulated/maintained at 20 °C by a water chilling system. The (Hitachi S-4300, Tokyo, Japan) FE-SEM apparatus was used to observe the microstructure evolution induced by DSR through electron backscattered diffraction (EBSD). Scanning electron microscopy (Hitachi S-4800, Tokyo, Japan) was employed to study the structural and coating morphologies. Assessments of corrosion protection involved conducting polarization tests in a 3.5 wt.% NaCl solution by using an electrochemical workstation (Gamry Interface 1000, Philadelphia, PA, USA). Further details can be found in the specified source [12].

## 3. Results and Discussion

### 3.1. Deformation of Al Sheet Alloy via DSR

**Figure 2:**
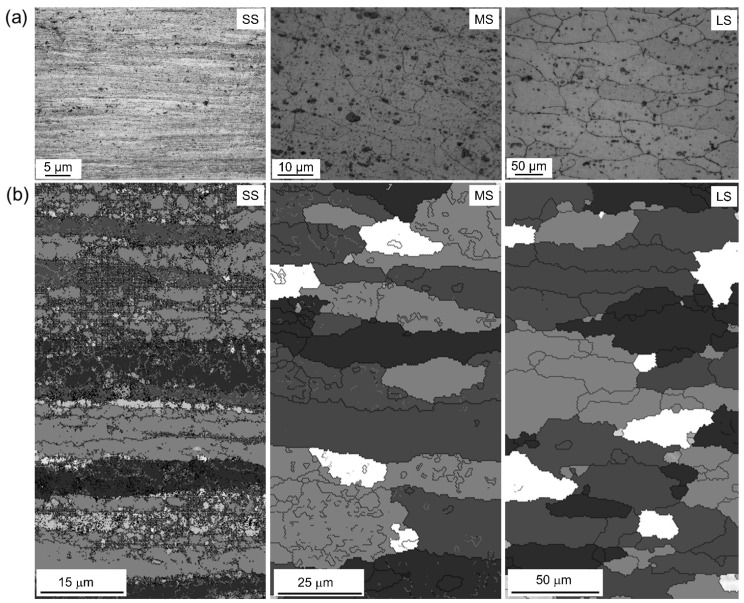
(**a**) Optical microscopy showing variation of grain size from samples prior to PEO after DSR followed by annealing at different temperatures and times, obtaining small-sized grain (SS), medium-sized grain (MS), and large-sized grain (LS). (**b**) Corresponding SEM-EBSD images of the samples. The figure depicts an ND-RD plane with the top area near to the upper roll and bottom area near to the bottom roll.

Figure 2 illustrates the (a) optical micrograph and (b) EBSD images associated with the microstructural characteristics that developed in the AA6061 alloy samples following deformation via DSR at a roll-speed-ratio of 1:4 (resulting in up to 75% reduction), followed by a subsequent annealing process at 25 °C (SS), 550 °C for 3h (MS), and 560 °C for 12 h (LS) to obtain the desired sizes of ~1 μm, ~25 μm, and ~55 μm. Based on our previous work, the evolution of the grain size (<1 µm) was obtained after four passes with ~74% reduction by means of the nucleation of high angle boundaries at the expense of low angle grain boundaries [13].

### 3.2. Voltage Response and Plasma Characteristics

**Figure 3:**
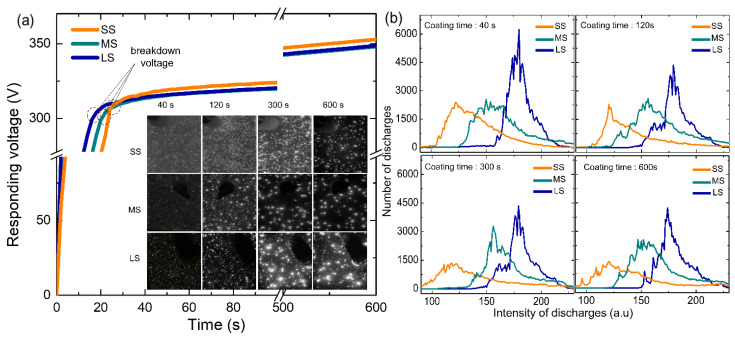
(**a**) Responding voltage vs. coating time curves of the samples with small-sized grain (SS), medium-sized grain (MS), and large-sized grain (LS). Inset showing the evolution of plasma discharges formed on the samples during PEO with respect to coating times of 40, 120, 300, and 600 s. (**b**) Curves showing the number and intensity of plasma discharges.

The initiation and behavior of plasma discharges, encompassing their size, density, duration, and intensity, play a pivotal role in governing the overall microstructures of coatings during the plasma electrolytic oxidation (PEO) process. Monitoring these discharge characteristics throughout PEO is essential for a comprehensive understanding of the coating’s microstructures and the evolving mechanisms of electrolyte species’ growth. Figure 3a shows the rms voltage responses of samples with three different grain sizes: small, medium, and large, which would be ascribed as bath A and bath B, respectively. Regardless of the grain size, Figure 1a reveals the identification of two distinct stages, determined by the rate at which voltage increased concerning processing time. During stage 1, the rapid increase in voltage adhered to Ohm’s law, attributed to the swift passivation of the metal substrate, subsequently leading to an elevation in electrical resistance. Until surpassing the breakdown voltage, the film underwent continuous growth. Upon exceeding this threshold, the barrier layer transitioned to a conductive state, causing the film’s barrier structure to become porous, as indicated by the gradual decrease in slope, denoted as stage II. The initiation of stage II was heralded by the emergence of minute, rapid sparks on the sample surface, accompanied by acoustic emission resulting from the breakdown of O_2_ gas.

From Figure 3b, the breakdown voltage recorded was higher for the SS sample (~315 V) as compared to that of the MS (~310 V) and LS (~308 V) samples, which was strongly associated with the initial grain size. The sparking voltage was found to correlate with the conductivity characteristics of both the electrolyte and the substrate materials [14]. The significant breakdown voltage observed in SS could be linked to its elevated resistivity attributed to the formation of a protective film upon contact with an electrolyte [15]. This connection between the high breakdown voltage and the resistive nature of the passive film aligned with findings by Ikonopisov et al. [14], suggesting that the substrate’s surface condition influenced the breakdown voltage. It is noteworthy to highlight that the time taken to reach the breakdown voltage decreased as the grain size increased. The time taken to reach the breakdown was recorded at 32 s for the SS sample, whilst this was longer for the MS and LS samples, at 22 and 27 s, respectively. Evaluating both the voltage and breakdown time, it is probable that the LS sample would necessitate the least amount of energy during the PEO process.

The inset of Figure 3a shows optical images of plasma discharges during PEO for all samples at specific coating times. The upper-side shows more homogeneous plasma discharges whereas the middle and bottom sides show more localized plasma discharges with relatively higher intensity. Homogeneous plasma discharges could signify a consistent voltage response, whereas localized discharges may lead to fluctuations in the voltage response. In terms of plasma intensity, the small-sized (SS) sample exhibited a comparatively more uniform and subdued discharge intensity during the initial and intermediate stages of the plasma electrolytic oxidation (PEO), as evidenced in Figure 3b. Conversely, the medium and high intensity regions displayed by the medium-sized (MS) and large-sized (LS) samples indicated a less uniform distribution throughout the entire PEO process.

Furthermore, these traits could potentially be linked to the attributes of its coating, including porosity and composition. Upon analyzing video imaging data, the high intensity of plasma discharges observed during the 5 and 10 min PEO processes in both the MS and LS samples might not be conducive to achieving a dense oxide layer due to the presence of plasma discharges exhibiting adverse behavior.

### 3.3. Morphologies and Composition of the Coating

**Figure 4:**
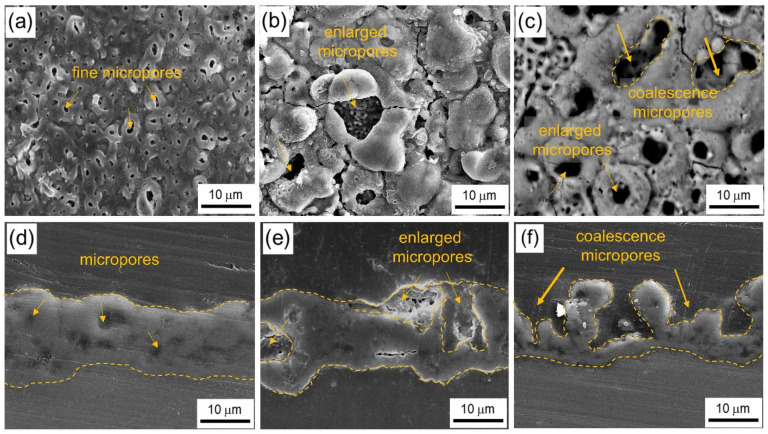
SEM images showing the surface morphologies of the coating formed on (**a**) SS, (**b**) MS, and (**c**) LS samples. Cross-sectional images showing a comparable coating thickness of (**d**) SS, (**e**) MS, and (**f**) LS samples. As the initial grain size of the Al alloy increases, both the pore size and the compactness of the coating decrease.

Figure 4a–f show surface morphologies and cross-sectional images of the samples after PEO. From Figure 4a–c, the microstructures show a typical porous surface of PEO coating, irrespective of the initial grain size. In general, the porosity decreased with decreasing grain size. It is noteworthy that the SS sample showed relatively fine micropores, while the MS sample started to develop enlarged micropores and the LS sample exhibited interconnected (coalescence) micropores. As evident from Figure 4a–c, the surface compactness of the SS sample was significantly altered from that of a larger grain. The surface morphologies were in line with the cross-sectional images shown in Figure 4d–f, where the SS sample showed better compactness than that of larger-grained samples. This was due to the presence of enlarged micropores and coalescence micropores in MS and LS samples, respectively.

On the other hand, the coating in the SS sample showed comparable thickness with the MS and LS samples of approximately ~10 µm. The comparable thickness, coupled with improved compactness, could suggest enhanced corrosion resistance. Previous works documented that the specimen featuring a greater coating thickness is expected to offer superior corrosion protection compared to the one with a thinner coating [2,12,16].

**Figure 5:**
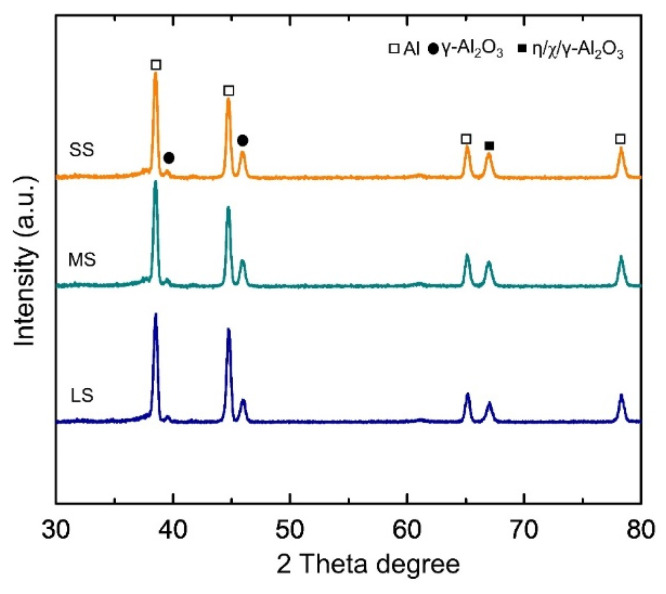
XRD pattern of all the samples after PEO, showing Al_2_O_3_ as the majority fraction.

To determine constitutive compounds that are present in the samples, XRD patterns in the range of 30~80 2θ degrees are shown in Figure 5. Most of the detected peaks correspond to γ-Al_2_O_3_ and metastable η/χ/γ-Al_2_O_3_. The detection of the aluminum peak occurred because the X-rays penetrated deeply during the analysis. The oxide layers were mainly composed of metastable Al_2_O_3_ (including η-Al_2_O_3_ (JCPDS #13-0373), χ-Al_2_O_3_ (CCD #00-004-0875)), and γ-Al_2_O_3_ (JCPDS #00-010-0425). The fraction of Al_2_O_3_ was found to be apparent, which was probably due to the sequential process involving dehydration and transformation reactions from the hydroxide form by means of high-energy plasma. The presence of Al_2_O_3_ as the main matrix of the coating suggested that the surface hardness of the coating would be expected to increase in value.

### 3.4. Microhardness of the Coating

**Figure 6:**
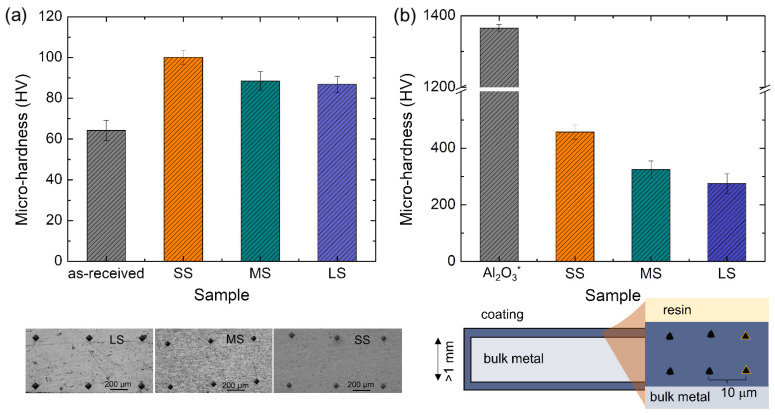
(**a**) Microhardness of as-received 6061-T6 Al alloys, deformed samples, and deformed-annealed samples; (**b**) nanohardness of PEO-coated samples compared to the hardness value of Al_2_O_3_* from a different study as reference [17].

Figure 6 shows the microhardness of the samples before and after the PEO process. As revealed by the graph in Figure 6a, the microhardness of the as-received Al alloy was approximately 64 HV. After four-pass severe plastic deformation (SS sample), the microhardness value increased to about 102 HV. The microhardness values decreased to ~88.5 and 86.8 HV when subjected to annealing at temperatures of 550 °C for 3 h and 560 °C for 12 h, respectively. From previous studies, it was documented that the hardness of Al_2_O_3_ was approximately 1000~1300 HV with a dense microstructure processed via centrifugal slip casting [17]. On the other hand, those having porous structures have hardnesses in the range of several hundred to a few thousand on the hardness scale (370~1010 HV) in the presence of YAG [18]. Al_2_O_3_ which is grown via chemical vapor deposition can reach the range of 2519 to 2947 HV [19].

In a study by Peng et al. [20], where the microhardness of their PEO-coated sample reached a value of 8 times higher than that of the uncoated 6061 Al alloy, their coating had greater thickness (~50 μm) and compactness due to the prolonged coating time of up to 60 min (6 times higher than that in the present study). They additionally proposed a correlation between the treatment duration and the microhardness of PEO coatings formed in the identical electrolyte, indicating that as the treatment time increases (from 30 min to 60 min), the hardness of the coating rises—thus, suggesting that greater PEO coating thickness corresponds to increased hardness. Moreover, the presence of α-Al_2_O_3_ in their results was believed to be the reason for the high value of microhardness, whilst the present study was dominated by γ-Al_2_O_3_ which is known for a lower hardness value [19,21]. Nevertheless, it was evident that the DSR process improved the microhardness value of the bulk sample and the use of PEO successfully improved the microhardness value of the surface. In the future, we can improve the hardness value of the surface by (i) increasing the compactness of the coating, (ii) enlarging the amount of hard-phase such as α-Al_2_O_3_, and (iii) increasing the coating thickness. The use of ultrasonic during PEO might also be imparted in future works, since it was documented that an ultrasonic wave can reduce porosity as well as increase coating compactness [12].

### 3.5. The Corrosion-Resistant Properties of the Coating

**Figure 7:**
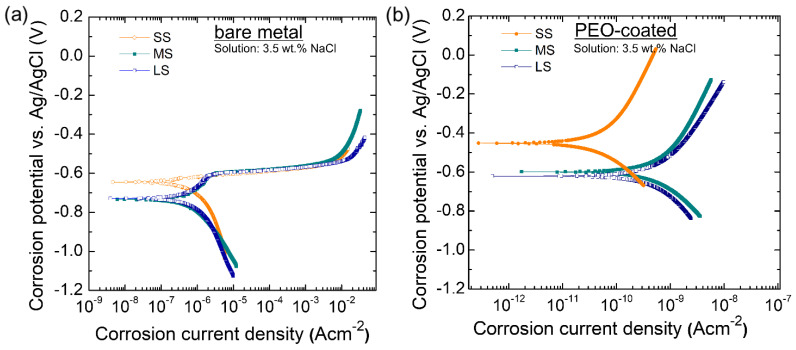
(**a**,**b**) Potentiodynamic polarization curves in 3.5 wt.% NaCl for the samples (**a**) before PEO (bare metal) and (**b**) after PEO (coated surface). Small-grained samples showed better protective capability.

The polarization curves of the samples prior to and after PEO are shown in Figure 7a,b. The electrochemical parameters encompassing current density of the corrosion (*i_corr_*), corrosion potential (*E_corr_*), and Tafel slope (*β_a_*/*β_c_*) for anodic/cathodic branches were obtained from the Tafel extrapolation whilst the polarization resistance (*R_p_*) values were estimated using the Stern–Geary equation as given in Equation (1) and the results are presented in Table 1.

(1) $$R_{p}=\frac{\beta_{a}\cdot \beta_{c}}{{2.303\;i_{corr}(\beta }_{a}+\beta_{c})}$$

It can be inferred from Figure 7a that in general, *E_corr_* values for the finer grain were shifted to a nobler region compared to those of coarser grain, which was probably due to the formation of passive film with better chemical stability. The results are in accordance with the documented work by Kim et al. [22] where Ti exhibiting a more finely tuned microstructure displays increased positive E_ocp_ values and extended critical time before corrosion failure. Kim et al. [22] proposed that employing high-ratio dual-step rolling to refine the grain in pure Ti enhanced its ability to resist corrosion by changing how the passivating oxide film grows. This was due to the fact that the grain boundary has higher energy than the grain interior; both corrosion and passivation thus preferred to occur in the grain boundary region [22]. In addition, more grain boundaries in nanocrystalline materials often lead to improved corrosion resistance by enhancing the kinetics of passivation, enabling the rapid formation of a stable protective layer.

Moreover, Balyanov et al. [23] documented that ECAP-processed ultrafine-grained titanium showcased superior corrosion resistance in 1 M HCl and 1 M H_2_SO_4_ solutions compared to coarse-grained Ti, evidenced by lower corrosion current densities, more positive corrosion potentials, decreased critical currents at passive potential, and the rapid formation of protective films at surface defects such as grain boundaries and dislocations, while impurity segregation at grain boundaries likely accelerated the corrosion process in coarse-grained Ti.

According to Ralston et al. [24], the mechanism of corrosion involving grain size might be two-fold. First, in the presence of some degree of passive film where *i_coor_* is low, the *i_corr_* tends to decrease with decreasing grain size. The main driving force for this mechanism relies on the grain boundary densities which control the passive film formation based on Equation (2) below:

*i_corr_* = A + B*d*^−0.5^ (2)

where A is the constant related to the environment, B is the constant of the inherent materials affected by alloying compounds, etc., and *d* is the grain size. Second, in the absence of the passive film, where the corrosion rate is high due to the direct contact of the surface and the corrosive electrolyte, the decrease of grain size (increased grain boundary density) leads to an increase in surface reactivity and *i_corr_*.

Due to the similar coating thickness and constitutive compounds of the coating layer, the microstructural features such as high coating compactness were taken into account for the alteration of corrosion behavior. Figure 7b shows that, in general, both the *E_corr_* and *i_corr_* of the samples shifted to the nobler region and lower corrosion current density, which is beneficial for improving anti-corrosion properties.

The increased density of grain boundaries in the small-sized sample could potentially influence the surface reactivity of the metal when exposed to the electrolyte [25]. During PEO, the high applied current on the sample accelerates passive film formation. Electrons, in electrical conduction, experience scattering along the grain boundaries [26], which slows their movement towards nucleation sites. However, as a fixed current density was applied, the total energy flow along the grain remains comparable. Despite each grain in the SS sample being subjected to a lower number of electrons, more sites are available for the nucleation of the passive film.

Owing to the porous structures grown via PEO, another surface treatment such as a selective induced electrolytic process with full control of porosity could be considered. However, it is worth noting that the selective laser induced electrolytic process, while effective, often entails higher costs and longer processing times [27].

## 4. Conclusions

The enhancement of a combination of both reliable and protective properties was achieved via differential speed rolling (DSR) and plasma electrolytic oxidation (PEO) on 6061 Al alloy by means of grain refinement and thick film formation assisted by plasma energy. The high hardness was obtained due to grain refinement (<1 μm) after four consecutive passages of severe plastic deformation. The high corrosion resistance arises from the presence of a thick and hard Al_2_O_3_ matrix of the PEO coating, improving the corrosion properties by up to one order of magnitude when a dense and thick coating is obtained. The hardness was recorded to increase from ~64 to ~102 HV in the presence of an Al_2_O_3_ layer. This collaboration results in customized materials suited for high-performance uses across various industries, blending improved bulk characteristics achieved through DSR with finely tuned surface qualities obtained from PEO.

## Figures and Tables

**Table 1:** Polarization resistance of the samples before PEO (bare) and after PEO (coated). These tests were carried out in a 3.5 wt.% NaCl solution, with measurements taken from −0.25 to 0.4 V concerning the open circuit potential.

Sample	*i_corr_* (A·cm^2^)	*E_corr_* (V)	*β_a_* (V/decade)	*β_c_* (V/decade)	*R_p_*
SS (bare)	1.07 × 10^−6^	−0.645	0.271	−0.510	7.18 × 10^4^
MS (bare)	1.34 × 10^−6^	−0.734	0.298	−0.320	5.90 × 10^4^
LS (bare)	3.47 × 10^−6^	−0.729	0.985	−0.904	5.00 × 10^4^
SS (coated)	7.58 × 10^−11^	−0.453	0.589	−0.346	1.25 × 10^9^
MS (coated)	9.74 × 10^−10^	−0.580	0.680	−0.771	1.24 × 10^8^
LS (coated)	1.05 × 10^−9^	−0.628	0.676	−0.410	1.06 × 10^8^

## Data Availability

Data are contained within the article.

## References

[1] Yerokhin A.L., Nie X., Leyland A., Matthews A., Dowey S.J. (1999). Plasma Electrolysis for Surface Engineering. Surf. Coat. Technol..

[2] Kaseem M., Fatimah S., Nashrah N., Ko Y.G. (2021). Recent Progress in Surface Modification of Metals Coated by Plasma Electrolytic Oxidation: Principle, Structure, and Performance. Prog. Mater. Sci..

[3] Amegadzie M.Y., Bishop D.P. (2020). Effect of Asymmetric Rolling on the Microstructure and Mechanical Properties of Wrought 6061 Aluminum. Mater. Today Commun..

[4] Hamad K., Ko Y.G. (2019). Continuous Differential Speed Rolling for Grain Refinement of Metals: Processing, Microstructure, and Properties. Crit. Rev. Solid State Mater. Sci..

[5] Vincze G., Simões F., Butuc M. (2020). Asymmetrical Rolling of Aluminum Alloys and Steels: A Review. Metals.

[6] Bahmani A., Kim W.J. (2020). Effect of Grain Refinement and Dispersion of Particles and Reinforcements on Mechanical Properties of Metals and Metal Matrix Composites through High-Ratio Differential Speed Rolling. Materials.

[7] Son I.J., Nakano H., Oue S., Kobayashi S., Fukushima H., Horita Z. (2008). Effect of Annealing on the Pitting Corrosion Resistance of Anodized Aluminum-Magnesium Alloy Processed by Severe Plastic Deformation. Mater. Trans..

[8] Osório W.R., Freire C.M., Garcia A. (2005). The Role of Macrostructural Morphology and Grain Size on the Corrosion Resistance of Zn and Al Castings. Mater. Sci. Eng. A.

[9] Wang P., Zhao J., Ma L., Cheng X., Li X. (2021). Effect of Grain Ultra-Refinement on Microstructure, Tensile Property, and Corrosion Behavior of Low Alloy Steel. Mater. Charact..

[10] Reshadi F., Faraji G., Baniassadi M., Tajeddini M. (2017). Surface Modification of Severe Plastically Deformed Ultrafine Grained Pure Titanium by Plasma Electrolytic Oxidation. Surf. Coat. Technol..

[11] Chung M.K., Choi Y.S., Kim J.G., Kim Y.M., Lee J.C. (2004). Effect of the Number of ECAP Pass Time on the Electrochemical Properties of 1050 Al Alloys. Mater. Sci. Eng. A.

[12] Fatimah S., Hazmatulhaq F., Sheng Y., Suhartono T., Oh J.M., Nashrah N., Kang J.H., Ko Y.G. (2023). Effect of Ultrasonic Frequency on Structure and Corrosion Properties of Coating Formed on Magnesium Alloy via Plasma Electrolytic Oxidation. Materials.

[13] Ko Y.G., Masood Chaudry U., Hamad K. (2020). Microstructure and Mechanical Properties of AA6061 Alloy Deformed by Differential Speed Rolling. Mater. Lett..

[14] Ikonopisov S. (1977). Theory of Electrical Breakdown during Formation of Barrier Anodic Films. Electrochim. Acta.

[15] Yerokhin A.L., Nie X., Leyland A., Matthews A. (2000). Characterisation of Oxide Films Produced by Plasma Electrolytic Oxidation of a Ti-6Al-4V Alloy. Surf. Coat. Technol..

[16] Lu X., Mohedano M., Blawert C., Matykina E., Arrabal R., Kainer K.U., Zheludkevich M.L. (2016). Plasma Electrolytic Oxidation Coatings with Particle Additions—A Review. Surf. Coat. Technol..

[17] Zygmuntowicz J., Miazga A., Konopka K., Kaszuwara W. (2016). Metal Particles Size Influence on Graded Structure in Composite Al_2_O_3_-Ni. Mater. Tehnol..

[18] Paneto F.J., Pereira J.L., Lima J.O., Jesus E.J., Silva L.A., Sousa Lima E., Cabral R.F., Santos C. (2015). Effect of Porosity on Hardness of Al_2_O_3_-Y_3_Al_5_O_12_ Ceramic Composite. Int. J. Refract. Met. Hard Mater..

[19] Ruppi S., Larsson A., Flink A. (2008). Nanoindentation Hardness, Texture and Microstructure of α-Al_2_O_3_ and κ-Al_2_O_3_ Coatings. Thin Solid Films.

[20] Peng Z., Xu H., Liu S., Qi Y., Liang J. (2021). Wear and Corrosion Resistance of Plasma Electrolytic Oxidation Coatings on 6061 Al Alloy in Electrolytes with Aluminate and Phosphate. Materials.

[21] Musil J., Blažek J., Zeman P., Prokšová Š., Šašek M., Čerstvý R. (2010). Thermal Stability of Alumina Thin Films Containing γ-Al_2_O_3_ Phase Prepared by Reactive Magnetron Sputtering. Appl. Surf. Sci..

[22] Kim H.S., Yoo S.J., Ahn J.W., Kim D.H., Kim W.J. (2011). Ultrafine Grained Titanium Sheets with High Strength and High Corrosion Resistance. Mater. Sci. Eng. A.

[23] Balyanov A., Kutnyakova J., Amirkhanova N.A., Stolyarov V.V., Valiev R.Z., Liao X.Z., Zhao Y.H., Jiang Y.B., Xu H.F., Lowe T.C. (2004). Corrosion Resistance of Ultra Fine-Grained Ti. Scr. Mater..

[24] Ralston K.D., Birbilis N., Davies C.H.J. (2010). Revealing the Relationship between Grain Size and Corrosion Rate of Metals. Scr. Mater..

[25] Lejček P. (2010). Grain Boundaries: Description, Structure and Thermodynamics. Grain Boundary Segregation in Metals.

[26] Kwapuliński P., Rasek J., Gierak Z. (1988). Scattering of Conductivity Electrons on Grain Boundaries in Metals. Phys. Status Solidi.

[27] Zhu H., Zhang M., Ren W., Saetang V., Lu J., Wu Y., Xu K., Liu Y., Zhang Z. (2024). Laser-Induced Localized and Maskless Electrodeposition of Micro-Copper Structure on Silicon Surface: Simulation and Experimental Study. Opt. Laser Technol..

